# Value of modified axial review radiograph in diagnosing calcaneal fractures

**DOI:** 10.1038/s41598-020-70460-w

**Published:** 2020-08-11

**Authors:** Zhe Guo, Bao-Hai Yu, Shu-Man Han, Lei Cao, Hui-Zhao Wu, Zhu-Zuo Zhang, Wen-Juan Wu, Bu-Lang Gao

**Affiliations:** Department of Radiology, The Third Hospital of Hebei Medical University, 139 Ziqiang Road, Shijiazhuang, 050051 Hebei Province China

**Keywords:** Bone, Bone imaging

## Abstract

To investigate the value of modified calcaneal axial radiograph—the horizontal calcaneal axial radiograph in diagnosing calcaneal fractures, patients who had acute calcaneal fractures or internal fixation were enrolled, and three groups were established, including the acute fracture group (n = 20), the internal fixation group (n = 20), and the healthy control group (n = 20). All the subjects had regular and modified calcaneal axial radiograph for comparison. In analysis of the results, all volunteers could have ankle dorsiflexion at different degrees. When the ankle was at 30º dorsiflexion for regular axial radiograph, the subtalar joint and the sustentaculum tali could not be clearly displayed. The calcaneus was elongated if the tube tilted in a larger angle but shortened if the tube titled in a smaller angle. When the ankle was at neutral (0º dorsiflexion) location with the tube tilting 45° cephalad or when the ankle was at 20° plantarflexion with the tube tilting 35° cephalad, the subtalar joint, sustentaculum tali, calcaneal body and internal and external calcaneal processes could all be clearly demonstrated. No significant difference (*P* = 0.79) existed in displaying the bony anatomical structures in regular compared with modified calcaneal axial radiography. For patients with acute calcaneal factures or with internal fixation, the modified calcaneal axial radiography could display more significantly clearly (*P* = 0.001) bony anatomical structures than the regular one. In conclusion, the modified calcaneal axial radiograph can be performed easily and can clearly show the bony structure of the calcaneus and surrounding bones without adding pain to the patients with calcaneal fractures.

## Introduction

In assessing calcaneal fractures with plain radiographs, surgeons usually use lateral and axial views for the diagnosis and treatment^1,2^, especially the axial view of plain radiographs^3^. Zhang et al.^3^ have studied the important role of the axial radiograph of calcaneus in diagnosing calcaneal fractures and found that the axial view is very useful in diagnosing patients with suspected calcaneal fracture especially in distinguishing intra-articular fractures. Using the axial plain radiograph of the calcaneus, Soeur and Remy^4^ even classified the calcaneal intra-articular fractures into three types: first-degree fractures being nondisplaced shear fractures with widening of the joint surface, second-degree including secondary fracture lines with a minimum of three pieces, and third-degree being highly comminuted. When taking axial radiographs of the calcaneus, the ankle is placed in maximum dorsiflexion so as to better demonstrate the calcaneal talus process, subtalar joint, calcaneal body, medial and lateral processes and other anatomical relationship. However, for patients with acute calcaneal fractures or internal fixation in calcaneal fractures, the ankle joint is limited in dorsiflexion/range due to pain, soft tissue swelling and some hemarthros, which will affect the calcaneal axial view radiography to clearly show the calcaneal joint. So, a modified position for axial radiography of the calcaneus is needed in acute situation. This study aimed to design a modified position for calcaneal axial view radiography—the horizontal calcaneal axial view which can be used for patients with limited ankle dorsiflexion/range so as to better evaluate the major anatomical structure of the ankle joint.

## Materials and methods

This study was approved by the ethics committee of the Third Hospital of Hebei Medical University with all patients or their legal guardians given their signed informed consent. All methods were performed in accordance with the relevant guidelines and regulations. Between November 2015 and March 2016, patients who had acute calcaneal fractures or internal fixation of calcaneal fractures were enrolled, and three groups were established, including the acute calcaneal fracture group without internal fixation or the fracture group (n = 20), the internal fixation group (n = 20), and the healthy control group (n = 20). For the fracture group, all patients had limited movement of the ankle joint with the angle formed between the horizontal level of the foot and the tibial shaft greater than 90°. There were 18 male and 2 female patients with an age range of 12–60 years (mean 36.35 ± 13.05) in this group (Table 1). Axial view radiography was performed on the left for 15 cases and on the right for 5 cases. For patients in the internal fixation group, the ankle joint also had different limitations in ankle dorsiflexion, including 15 male and 5 female patients with an age range of 25–61 years (mean 36.35 ± 12.32). Axial view radiography was performed in 10 patients on the left and in the rest on the right. For the healthy control group, 8 male and 12 female volunteers were enrolled with an age range of 12–65 years (mean 38.20 ± 14.01), and the axial view radiography was taken in 13 cases on the left and 8 cases on the right, including one case who had axial radiography on both sides (Table 1). All the subjects had both the normal axial view radiography and modified axial view radiography for comparison.

**Table 1:** Demographic data of patients in three groups.

Parameters	Fracture group (n = 20)	Internal fixation (n = 20)	Healthy control (n = 20)
M:F	18:2	15:5	8:12
Left: right	15:5	10:10	13:8
Age	36.35 ± 13.05	36.35 ± 12.32	38.20 ± 14.01

For regular axial view radiography, the volunteers were laid supine on the X-ray table with ankle dorsiflexion in different angles. The plate detector or cassette was placed perpendicular to the table and in close contact with the plantar surface, and at the ankle location of 30° dorsiflexion, neutral (0° dorsiflexion) location, and 20° plantarflexion , the central line of the X-ray tube projected 1 cm below the posterior malleolus for radiography. Then, the angle formed between the calcaneal long axis and the plantar surface was measured for deciding the X-ray tilting angle and major bone structures.

**Figure 1:**
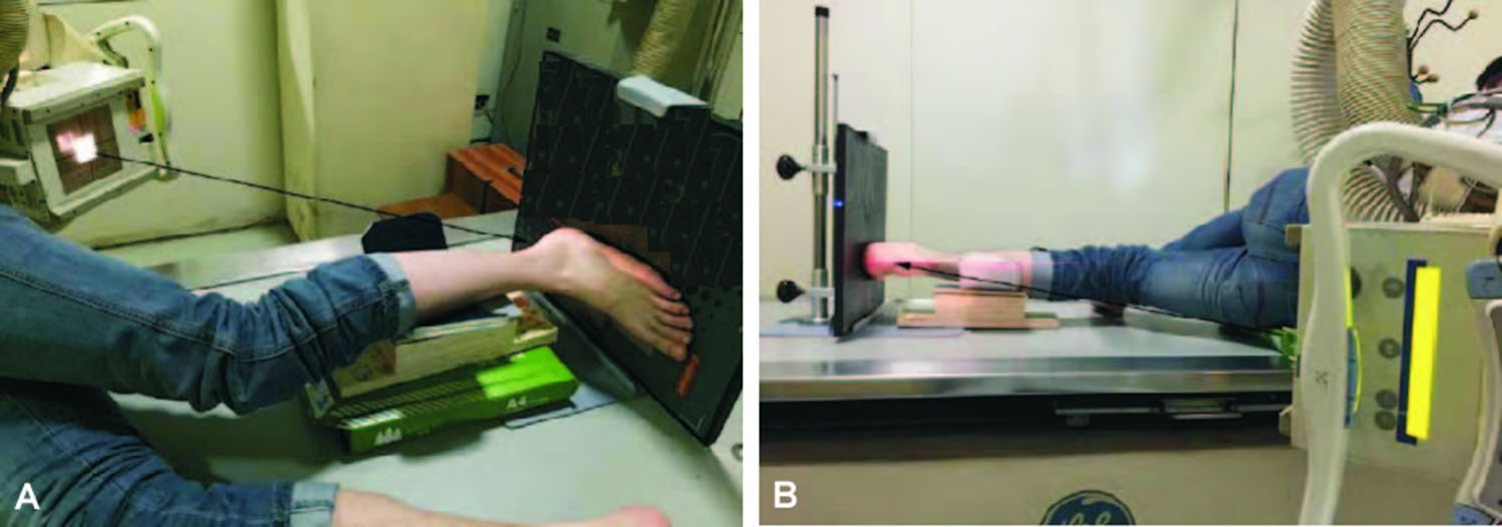
Modified horizontal calcaneal axial radiography from different perspectives.

The modified axial view radiograph was taken with the patients being laid on the side, the target tibia was put on a 5 cm high bracket, and the foot plantar surface was put in close contact with the cassette or the X-ray plate detector which was perpendicular to the table (Fig. 1). The X-ray tube was titled towards the plantar surface based on the angle between the calcaneus long axis and the plantar surface. If the angle between the calcaneal long axis and the plantar surface was great, the tilted angle of the X-ray tube was great or vise versa. The titled angle of the tube was between 40° and 45°. The central line of the X-ray was projected at 1 cm below the posterior malleolus.

All the subjects in the three groups would have the normal and modified axial view radiography for comparison of major structures of the ankle anatomy including the subtalar joint, sustentaculum tali, calcaneal body and internal and external calcaneal processes. The evaluation criteria were set as 0 for complete inability to display the ankle joint, 1 for displaying less than 30% of the ankle joint, 2 for displaying 30–90% of the joint and 3 for clear demonstration of the joint^5^. Two radiographers blinded to the clinical data of the radiographs evaluated the two axial view radiographs and gave proper scores; when in disagreement, a third radiologist would be involved for reaching a consensus.

### Statistical analysis

The SPSS 19.0 (IBM, Chicago, IL, USA) for windows was used for analysis. Enumeration data were tested with the Chi-square test. The *P* value of < 0.05 was set as statistically significant.

## Results

**Figure 2:**
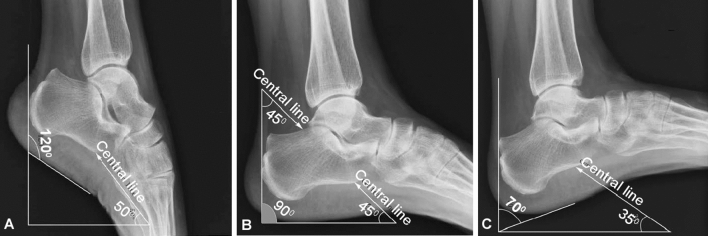
Calcaneal radiography in different positions in a male volunteer (28 years of age). (**A**) When the ankle was at 30° dorsiflexion and the tube tilts up to 50° cephalad, the tarsus and talus cannot be clearly demonstrated because they are overlapped with the subtalar joint and the sustentaculum tali. The calcaneus is elongated. (**B**) When the ankle was at the neutral location (0° dorsiflexion) and the tube tilts 45° cephalad, the tarsus, talus, subtalar joint, and sustentaculum tali can all be clearly shown. The calcaneal body is demonstrated in normal ratio with normal width and length. (**C**) When the ankle was at 20° plantarflexion and the tube tilts 35°, the tarsus, talus, subtalar joint, and sustentaculum tali can all be clearly displayed. The width and length of the calcaneus are normal.

For regular calcaneal axial view radiography, all the 20 volunteers could move the ankle freely at different degrees of dorsiflexion or plantarflexion. When the ankle was at 30° dorsiflexion for regular axial radiograph, the subtalar joint and the sustentaculum tali could not be clearly displayed at none of the three degrees of 45°, 50° and 55° by which the tube tilted cephalad (Fig. 2A). The calcaneal body was elongated if the tube tilted in a larger angle but shortened if the tube titled in a smaller angle. When the ankle was at the neutral (0°) location with the tube tilting 45° cephalad (Fig. 2B) or when the ankle was at 20° plantarflexion with the tube tilting 35° cephalad (Fig. 2C), the subtalar joint, sustentaculum tali, calcaneal body and internal and external calcaneal processes could all be clearly demonstrated (Fig. 2B). No significant difference (*P* = 0.79) existed in displaying the anatomical structures of the ankle joint in regular compared with modified calcaneal axial radiography.

**Figure 3:**
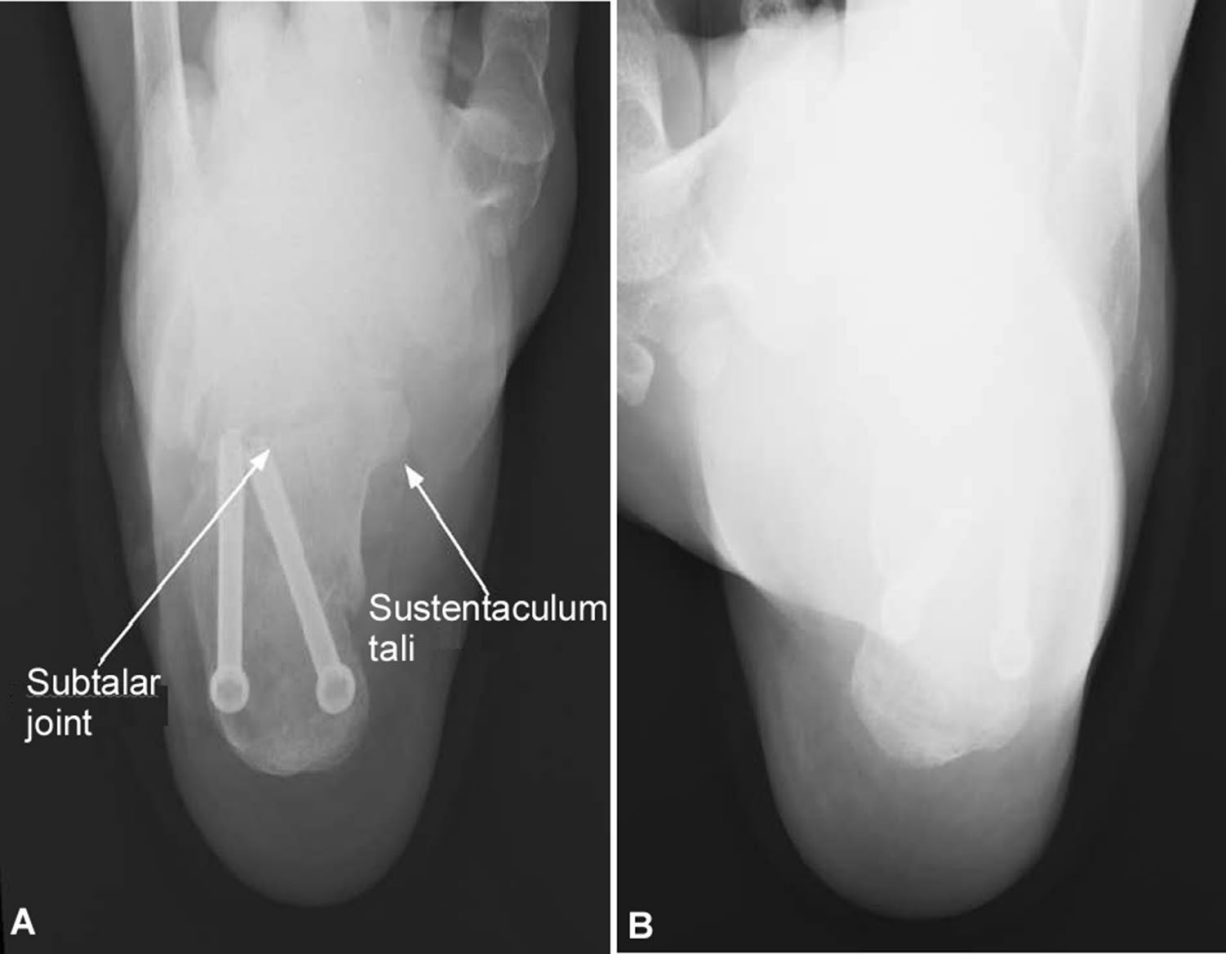
A male patient (30 years of age) had internal fixation for calcaneal fracture. (**A**) Modified horizontal calcaneal axial radiograph can clearly show the subtalar joint and sustentaculum tali. The calcaneus is without deformation. (**B**) Regular calcaneal axial radiograph cannot clearly demonstrate the subtalar joint or the sustentaculum tali.

For patients with acute calcaneal factures or with internal fixation for calcaneal fractures (Fig. 3), the ankle was limited in dorsiflexion/range, and the modified calcaneal axial radiography could display more significantly clearly (*P* = 0.001) the anatomical structures of the ankle joint than the regular axial radiography (Tables 2, 3).

**Table 2:** Display of ankle joint in regular compared with modified axial radiograph in acute calcaneal fractures.

	Normal ratio (1:2) between the width and length of calcaneus (%)				Abnormal ratio between the width and length of calcaneus (%)			
	SJ	ST	Calcaneus	CP	SJ	ST	Calcaneus	CP
Regular	25	20	10	0	0	10	15	70
Modified	25	65	85	30	75	80	90	100

Regular, regular calcaneal axial radiograph; Modified, modified calcaneal axial radiograph. SJ, subtalar joint; ST, sustentaculum tali; CP, calcaneal processes.

**Table 3:** Display of ankle joint in regular compared with modified axial radiograph in patients with internal fixation for acute calcaneal fractures.

	Normal ratio (1:2) between the width and length of calcaneus (%)				Abnormal ratio between the width and length of calcaneus (%)			
	SJ	ST	Calcaneus	CP	SJ	ST	Calcaneus	CP
Regular	25	10	0	0	0	20	20	65
Modified	15	45	80	35	75	90	100	100

Regular, regular calcaneal axial radiograph; Modified, modified calcaneal axial radiograph. SJ, subtalar joint; ST, sustentaculum tali; CP, calcaneal processes.

## Discussion

In this study investigating the value of modified calcaneal axial radiograph—the horizontal calcaneal axial radiograph in diagnosing calcaneal fractures, it was found that the modified calcaneal axial radiograph can be performed easily and can clearly show the bony structure of the calcaneus and surrounding bones without adding pain to the patients with calcaneal fractures.

The calcaneal fractures are problematic and present many obstacles to surgeons because of the irregular bony anatomy, complicated joint relationship, and dedicated soft tissue envelope^6^. To reduce post-operative sequalae and complications, proper diagnosis and good display of bony anatomy is helpful. In patients with calcaneal fractures but with negative results of X-ray examinations, spiral computed tomography (CT) scanning can detect the occult fractures and provide imaging bases for clinical treatment and judicial identification^7^. Moreover, misdiagnosis of these calcaneal fractures may lead to skin necrosis and lifelong disability^8^. This is why correct diagnosis of calcaneal fractures is of paramount importance in these calcaneal fracture patients with limited talocrural motion which poses great difficulties in taking proper calcaneal views for observing the anatomical structures. Lateral and axial calcaneal radiographs are the standard plain radiograms for evaluating calcaneal fractures. Lateral radiography has gained popularity in this aspect^1,2^ and is helpful in assessing two typical indexes of Bohler and Gissane angles^2,3^. The calcaneal axial radiograph is useful in evaluating suspected calcaneal fracture especially the intra-articular ones^3^. Good axial radiographs can clearly demonstrate the subtalar joint, sustentaculum tali, calcaneal body and internal and external calcaneal processes, thus playing a very important role in treating calcaneal fractures.

When the ankle is at plantarflexion in regular calcaneal axial radiography in normal volunteers, the distal end of the tibia will move forward, and the subtalar and ankle joints and the tarsal bone will not be overlapped by other bones. In this case, the calcaneal axial radiograph with the tube tilting 35° cephalad will show clearly the sustentaculum tali, subtalar joint and calcaneus. When the ankle is at dorsiflexion, the distal end of the tibia will move backward and overlap with the frontal part of the calcaneus and tarsal bone, and the subtalar joint, sustentaculum tali, ankle joint and tarsal bone will be overlapped with one another. In this case, the tube has to tilt a greater angle cephalad (50°–55°) so as to offset partial overlap of the bones for clear demonstration of the joints. If the tilting angle of the tube continues to increase, more of the subtalar joint will be overlapped, the calcaneus will be elongated, and the major anatomical structure of the joints will not be clearly demonstrated in the calcaneal axial radiograph. Therefore, in regular calcaneal axial radiograph, the dorsiflexion or plantarflexion degree of the ankle will determine the clarity of the calcaneus being demonstrated.

The standard lateral and axial views are well known and can be combined to evaluate the calcaneal fractures and postoperative reduction. Less well known are the oblique views by Brodén^9^, Isherwood^10^ and Anthonsen^11^. These oblique views have been proven useful in visualizing the extent of the fracture lines in the posterior facet after trauma, but almost all of these views have been replaced by CT in the last 2 decades^12^. CT scanning is three dimensional, which is critical to visualize the complex anatomical structures of intra-articular calcaneal fractures, and the Sanders classification system used for classifying intra-articular calcaneal fractures is based on CT three-dimensional scanning^13^. With the multiplanar reconstructions and volume rendering reconstructions, CT scanning permits classification of intra-articular calcaneal fractures, better evaluation and characterization of fracture lines and displacement of bone fragments. However, compared with CT, the plain radiograph is more readily available and reduces the risks of radiation significantly. Soft tissue injury and calcaneal fracture limit talocrural motion and ankle dorsiflexion, resulting in the difficulty to obtain the standard axial calcaneal radiographs and a reduction in the ability to visualize the anatomic structures. The modified axial calcaneal view in our study is actually a horizontal ski-view or harris-beath view, which was modified in order to reduce the effect of soft tissue injury after acute trauma (fracture) on standard calcaneal axial imaging.

In regular calcaneal axial radiography for patients with acute calcaneal fractures or internal fixation, even the ankle is elevated and the tube is tilted, the subtalar joint cannot be displayed clearly, and the calcaneus bone will be elongated in image, which does not satisfy the need for correct diagnosis. However, the modified axial view can clearly show the degree of varus, displacement and calcaneal tubercle shortening, calcaneal width, subtalar articular surface, and sustentaculum tali. It is mainly to help making the operation plan and evaluating the reduction effect after operation (if the ideal anatomical reduction has been achieved). The normal length to width ratio of the calcaneus is 2:1, and the calcaneus in some patients with calcaneal fractures will be shortened or widened and will have varus or displacement after fracture. The axial calcaneal view can be used to evaluate whether the width and length of calcaneus and its alignment have been restored in operation. This modified calcaneal axial view can be performed easily and can clearly show the bony structure of the calcaneus and surrounding bones without adding pain to the patients with calcaneal fractures. There are some advantages with the calcaneal axial radiograph. A lateral position is required with no need of additional ankle movement or elevation of the ipsilateral leg. The tube can project from above the calcaneal processes so as to decrease overlap of surrounding bony structures, resulting in the normal length of the calcaneus in image. The tube may tilt to a certain degree based on shape of the calcaneal bone. The axial radiograph view can be taken for both left and right calcaneus in one position.

However, some researchers suggested that the calcaneal axial radiography may be valueless because this radiography poses additional radiation to the patient and was difficult to obtain in routine evaluation and diagnosis of calcaneal fractures^14^. Zhang et al.^3^ conducted a study to test the value of axial view in diagnosing calcaneal fracture by introducing an angle Z, and their study confirmed that calcaneal axial radiography is useful in diagnosing patients with suspected calcaneal fracture, especially in distinguishing intra-articular fractures and selection for computed tomographic scan. The introduction of the angle Z enables a clear view of the axial radiograph for intra- and post-operative evaluation of calcaneal fractures. Our study revealed that the modified calcaneal axial radiograph can be easily obtained without adding additional pain to the patients and can clearly demonstrate the bony structure of the calcaneus and surrounding bones.

In summary, the modified calcaneal axial radiograph can be performed easily to clearly show the bony structure of the calcaneus and surrounding bones without adding pain to the patients with calcaneal fractures.
